# *Vaping Endgame*: cultural approaches and preliminary findings of an adapted vaping pilot intervention for American Indian youth

**DOI:** 10.1093/tbm/ibag053

**Published:** 2026-08-22

**Authors:** Lizbeth Becerra, Guadalupe Ramos-Plascencia, Sarah Keller, April Go Forth, Lisa Craig, Jennifer B Unger, Claradina Soto

**Affiliations:** Department of Population and Public Health Sciences-Keck School of Medicine, University of Southern California, Los Angeles, CA, United States; Department of Population and Public Health Sciences-Keck School of Medicine, University of Southern California, Los Angeles, CA, United States; Department of Population and Public Health Sciences-Keck School of Medicine, University of Southern California, Los Angeles, CA, United States; Resources for Indian Student Education, Inc, Alturas, CA, United States; Resources for Indian Student Education, Inc, Alturas, CA, United States; Department of Population and Public Health Sciences-Keck School of Medicine, University of Southern California, Los Angeles, CA, United States; Department of Social Medicine, Population, and Public Health, University of California, Riverside, Riverside, CA, United States

**Keywords:** vaping, pilot interventions, cultural adaptation, American Indians, Community-Based Participatory Research, holistic approach

## Abstract

**Background:**

American Indian (AI) youth report disproportionately high e-cigarette use (16%), twice that of Non-Hispanic White youth (8%). However, culturally tailored vaping prevention interventions remain. Preliminary findings from the pilot assessment of *Vaping Endgame* (VE), an AI culturally adapted vaping prevention curriculum developed through a community-informed process, highlights pre–post outcomes, participant acceptability, and satisfaction.

**Methods:**

Using Community-Based Participatory Research and Positive Youth Development principles, the VE curriculum was adapted from the Intervention for Nicotine Dependence: Education, Prevention, Tobacco, and Health and included Medicine Wheel elements. AI students participated in the 5-day VE program. Pre–post assessment included knowledge, attitudes, perceived behavioral control, intentions toward e-cigarette use, and readiness to change vaping behaviors. Feedback from participants and Youth Advocate Team (YAT) members assessed acceptability and satisfaction of the curriculum.

**Results:**

Of 63 eligible AI students (7–12 grade), 32 participated in the VE pilot intervention (50.8% participation; 100% retention). Participants had a significant increase in knowledge of vaping harms (100% vs. 88%), commercial tobacco marketing tactics toward youth (94% vs. 97%), and ceremonial tobacco use (6% vs. 16%). Their perceived behavioral control against vaping significantly increased (*r* = −0.50, *P* < .001). Students rated the curriculum and facilitator (average 8.8/10). YAT members described pride contributing to the VE program.

**Conclusion:**

Preliminary outcomes showed changes in knowledge and perceived behavioral control of vaping, and high satisfaction of the VE program. To our knowledge, this is the first culturally responsive vaping curriculum developed and assessed for AI youth, suggesting potential for broader delivery.

Implications **Practice:** Schools and after-school programs serving AI youth should consider culturally tailored vaping prevention curricula that incorporate perspectives and community input to center relevance and engagement for AI youth.

**Policy:** School administrators and educators should adopt a health justice approach for vaping that addresses inequities, provides holistic and supportive interventions, and elevates the community as leaders.

**Research:** Researchers may consider the use of feasibility study frameworks to evaluate the effectiveness of culturally adapted prevention interventions among AI youth that incorporate community input leadership among youth.

## Introduction

American Indian (AI) youth report high prevalence of commercial tobacco and e-cigarette use [1, 2], indicating an urgent public health disparity. Although AI youth represent only 1% of the national student population [1], their current (past 30 days) commercial tobacco product use (including e-cigarette vaping) doubled to 16% between 2023 and 2024, which is twice the prevalence of Non-Hispanic White youth (8%) [2]. National data further show the prevalence of e-cigarette use among AI middle and high school youth (11.5%) surpasses that of all other youth groups (7% Black/African American, 6% Non-Hispanic White, 6% Hispanic/Latino, and 7% Multi-racial) [2].

Electronic cigarettes, or “vape” products, present a pervasive and evolving public health issue. Nearly all e-cigarette liquids contain nicotine, which is addictive and adversely impacts normal brain development in adolescents, particularly in areas responsible for learning, mood regulation, and impulse control [3, 4]. Additionally, potent carcinogens that are emitted through the non-combustible vapor are associated with lung injury (hypoxia), neurological disorders (e.g. seizures, tremors, syncope), and cardiovascular disease (e.g. hypertension, dyslipidemia, tachycardia) [5].

Traditional or ceremonial tobacco holds deep cultural and spiritual significance in AI communities [6]. For thousands of years, it has been used as a sacred medicinal plant in ceremonial or traditional practices that include offerings, healing, and spiritual cleansing [6, 7]. However, the colonization and commercialization of tobacco by European settlers have disrupted these sacred practices, turning tobacco into a commercial product and contributing to its misuse as a recreational substance [8]. Despite these disruptions, AI people remain strong and resilient, continuing to honor and use traditional tobacco for its original spiritual purpose today. At the same time, the commercial tobacco industry has been invasive and strategic, targeting AI communities with advertising and introducing new products like e-cigarettes directly into AI reservations, which have been falsely marketed as a safer alternative [9]. These marketing tactics have blurred the lines between traditional and commercial tobacco use, making it essential for AI youth to learn and understand the difference between the two.

The American Lung Association’s (ALA) Intervention for Nicotine Dependence: Education, Prevention, Tobacco, and Health (INDEPTH) program was used as the foundation to adapt and develop our vaping curriculum. INDEPTH is a four-lesson program, grounded in the Transtheoretical Model (TTM), that focuses on vaping prevention and serves as an alternative to suspension in school settings [10]. INDEPTH’s evaluation indicated short-term successes among participating youth: a 9% increase in awareness of tobacco’s impact, a 15% significant increase in awareness of nicotine dependence, and a 7% boost in confidence to quit e-cigarettes [10, 11]. Programs such as INDEPTH offer an evidence-informed foundation to prevent and address nicotine use in the general youth population [10, 12–16]; however, INDEPTH has not been culturally adapted to incorporate AI cultural context and traditional tobacco values. Only one pilot intervention for indigenous children has applied an indigenous framework called the Medicine Wheel, which is used to emphasize the circular phases of life [17]. This study showed preliminary reductions in smoking intentions among Aboriginal children, though its focus on cigarettes only, and Canadian fourth graders, limits applicability to AI adolescent vaping interventions [17].

Community-Based Participatory Research (CBPR) and Positive Youth Development (PYD) approaches have been used for youth focused e-cigarette prevention interventions [12]. CBPR and PYD approaches involve engaging youth in advisory boards, advocacy, and leadership roles in partnership with educators and researchers ensuring that community perspectives are integrated, well-represented, and that findings accurately reflect AI communities’ contexts [18]. Among AI youth, PYD’s five C’s (competence, confidence, character, and caring/compassion) and culturally specific approaches have reduced the associations between risk factors and risky behaviors by strengthening cultural identity, connectedness, and resilience [19, 20]. The scarcity of culturally relevant vaping interventions for AI youth emphasizes the urgent need to adapt existing prevention programs using CBPR and PYD principles to better reflect Indigenous worldviews and cultural strengths, while incorporating theoretical frameworks for potential behavior change and tobacco marketing assessment.

### Aims and purpose

This article presents descriptive and preliminary findings from a pilot intervention delivery of the *Vaping Endgame* (VE) curriculum among AI middle and high school students. Adapted from the ALA INDEPTH program using Medicine Wheel principles and developed in partnership with a Community Advisory Board (CAB) and Youth Advocacy Team (YAT), the curriculum evolved from an alternative-to-suspension model into a broader prevention approach for vaping. Given the pilot design and small sample size, our preliminary findings aim to (i) describe the AI cultural adaptation process of the VE curriculum, (ii) assess preliminary pre–post outcomes, and (iii) provide AI student participant’s and YAT member’s satisfaction and acceptability of the pilot intervention program to inform future vaping intervention development among AI youth.

## Methods

### Study design and setting

The study was made possible by a collaboration between the University of Southern California (USC) and Resources for Indian Student Education (RISE), which is one of 23 American Indian Education centers throughout California that provides mentorship and instrumental support to AI students living in rural Modoc County, located in Norther California. RISE provides mentorship and academic support to AI youth and is highly respected and trusted in the local Tribal community. With RISE’s longstanding community leadership and connections, the research team convened a CAB and YAT to oversee and support the cultural adaptations of the vaping pilot curriculum and intervention [18]. While this study was not designed as a formal feasibility study, we report the AI student participant’s and YAT member’s acceptability and satisfaction to inform on insights of this pilot study for future vaping interventions among AI youth. Tribal and USC’s Institutional Review Boards approved this study and did not deem it as a clinical trial.

### Participant recruitment

AI student participants were recruited into the pilot intervention VE from two school districts in Modoc County, which includes combined junior and senior high grade levels where AI youth represent ∼6% of the population [21, 22]. Given the small, rural population in this region, middle and high schools are consolidated into one school. Student eligibility criteria included: (i) be currently enrolled in a Modoc County middle or high school, (ii) be between the ages of 13 and 19, and (iii) self-identification as an AI. While our eligibility criteria are inclusive of both AI and Alaska Native youth, the community we partnered with in Modoc County only identifies as AI people. A total of 63 eligible AI students were identified through RISE’s staff and invited to an informational meeting at each school during a homeroom period regardless of vape use. Participation required written assent onsite and parent/caregiver consent packets completed and returned in-person by the first pilot intervention lesson. Student participants received a $25 gift card for each session attended.

### Procedures

#### VE pre–post survey assessment

AI student participants completed a baseline pencil and paper survey on a Friday, prior to the beginning of the 5-day consecutive lesson pilot intervention, and a post-survey at the end. Participants received a $25 gift card for completing the baseline and post-assessment, respectively.

#### Curriculum adaptations for VE

CBPR and PYD frameworks guided the early development of the pilot intervention to emphasize youth strengths, resilience, and emotional, cognitive, and relational development as key pillars to adolescent well-being [20, 23]. Qualitative data from youth focus groups were collected from the CAB and YAT members one year prior to delivering the pilot intervention [18]. Their qualitative input informed the pilot intervention, including the theoretical framework, curriculum content, cultural adaptations, and pre–post survey measures to align with AI values and lived experiences [18]. While initially conceptualized as an alternative to suspension, the pilot intervention evolved into a prevention pilot program. Many of the youth engaged in the pilot phase were non-vape users, and the CAB and YAT members emphasized the importance of centering the VE program in AI perspectives so that it would resonate with all AI youth, regardless of vape use.

INDEPTH’s four-lesson program focuses on commercial tobacco knowledge, its harmful effects, safer alternatives, and skills to resist social influence [10], which informed the VE curriculum. The pilot intervention was grounded in the Indigenous Medicine Wheel framework [24–26], a four-quadrant circle that is used to strengthen health education, mental health, social work, science and research. Indigenous cultures emphasize the Medicine Wheel to demonstrate the circular or cyclical phases of life, drawing from elements in nature (e.g. sun, flowers, seasons, and tree trunks) [26].

Adaptations from the Medicine Wheel included the integration of Native imagery, symbols, and teachings into each VE lesson in combination with PYD’s five C’s (competence, confidence, character, and caring/compassion) [20, 23]. The curriculum emphasized a holistic approach of healthy alternatives for preventing the initiation and managing vaping and commercial tobacco cravings, while strengthening students’ perceived behavioral control to resist offers from peers. A new first lesson was created to ground the VE curriculum in Native experiences and storytelling. The cultural and PYD adaptations resulted in a five-lesson curriculum: (i) build relational capacity through connection and storytelling (new lesson); (ii) build confidence in sharing their story about vaping with others with a focus on education about vapes; (iii) build self-advocacy in the midst of addictive behaviors and lay the groundwork for volition; (iv) rebuild their story in ways that support a healthy lifestyle, addressing chronic and current stressors; and (v) build community advocacy and celebrate transformation. Additionally, the curriculum covered topics such as self-discovery, expressing emotions through cultural symbolism, embracing AI identity, recognizing habits and stressors, and developing a collective vision for community well-being. For example, the students participated in an activity where they wrote down one alternative to vaping for different feelings (such as sadness, stress, and social pressure), shared their ideas with peers, and identified other feelings that may trigger vaping or other risky behaviors. To reinforce these teachings, student participants were given a stone with symbols representing the Medicine Wheel’s four directions for their medicine bag—a small pouch-like bag used to hold meaningful items. Table 1 presents the adaptations and the Medicine Wheel cultural elements used to develop the VE pilot intervention curriculum.

**Table 1:** Adaptations from the Medicine Wheel for the *Vaping Endgame* pilot intervention curriculum.

Core lesson components	Cultural elements and adaptations from Medicine Wheel	Example activities
**L.1 Program introduction and orientation** Understand INDEPTH program. Become familiar with requirements. Get acquainted with each other. Identify reasons for tobacco use.	Native American facilitator to enhance cultural alignment and trust. Program introduction centers Medicine Wheel teachings to emphasize interconnectedness of health, identity, and personal choice. Eagle animal guide narrative used to introduce decision-making and position youth as authors of their own health stories.	Youth introduce themselves and establish group norms. Initiation of personal medicine pouch that youth assemble across sessions, using symbolic stones that represent their commitment to health.
**L.2 Education about vaping and community context** Increase awareness of vaping risks and misinformation. Explore social influences on tobacco use.	Teaching through storytelling. Group-based reflection to examine vaping messages. Emphasis on sharing and dialogue rather than lecture-based instruction. Mouse animal guide used to explore emotional awareness.	Anonymous story-sharing circle where youth reflect on life experiences. Explore vaping messages to identify misinformation.
**L.3 Understanding addiction** Understanding nicotine dependence. Identify triggers and assess personal use patterns.	Holistic framing of addiction across Medicine Wheel domains (physical, mental, emotional, and spiritual). Bear animal narrative used to illustrate how addictive behavior can shift priorities and reduce personal agency. Acknowledge community support in gaining personal agency over health decisions.	“Defining My Why” worksheet to reflect on underlying motivations and personal goals. Youth calculate and discuss the costs of vaping and its impacts.
**L.4 Coping and alternatives** Understand cravings and develop strategies to cope. Identify healthier alternatives to vaping.	Cravings explored through the Medicine Wheel (mental, physical, emotional, spiritual). Coping strategies highlight resiliency, and community support. Buffalo animal narrative used to illustrate how repeated choices create patterns and how alternative pathways can be chosen.	“This or That”: group activity highlighting everyday decision-making and social influence. Activity to match emotional triggers with healthy coping strategies.
**L.5 Future and commitment to change** Reflect on learning. Develop action plan for behavior change. Strengthen commitment to cessation.	Future planning framed through volition to maintain balance across Medicine Wheel components. Lesson reflection and sharing personal goals. Identify role models, mentors, or supportive resources.	Mantra stone selection to represent self-commitment and affirmation. Reflection on completed medicine pouch as a resource.

#### VE pilot intervention delivery

*Vaping Endgame* was piloted over 5 days during a 50-min class period. This sequence format and setting was approved by school administration to increase accessibility and engagement. The curriculum was delivered by an AI facilitator from RISE who was not involved in the cultural adaptation process to minimize potential bias.

#### Recruitment and retention rate

Recruitment and participation were documented with the support of RISE staff, who provided a list of all eligible AI students. Subsequently, the overall recruitment rate was calculated based on consent form counts, while retention was determined using daily paper-based sign-in sheet counts.

### Measures

#### Demographics

Demographic questions for VE participants were intentionally limited to school name, gender, and grade level at the request of RISE. This decision reflected their commitment to protecting student participant’s confidentiality and avoiding the collection of unnecessary data.

#### E-cigarette use

To measure e-cigarette use (vaping) and other commercial tobacco product use, the survey items from INDEPTH’s pre–post survey was used with permission from the ALA [10, 27]. The survey included questions like, “If you vape, what type of vape do you use most?” with a range of e-cigarette device options (e.g. disposable, pre-filled pods) and “Which of these nicotine/commercial tobacco products have you used in the last 30 days?” (e.g. no use, e-cigarettes, chewing tobacco).

#### Knowledge of nicotine addiction and commercial tobacco marketing

Knowledge sample questions adapted from the National Youth Tobacco Survey (NYTS) [16] included: “Once someone starts using nicotine/commercial tobacco products (cigarettes, vapes, JUUL, snuff, chew, dip, hookah, etc.), do you think it becomes difficult for them to quit?” and “Do you think tobacco companies use bright colors and flavors (candy, fruit, mint) to get youth to use nicotine/commercial tobacco products (cigarettes, vapes, JUUL, snuff, chew, dip, hookah, etc.)?,” which offered the following answer options: (i) Definitely not, (ii) Probably not, (iii) Probably yes, (iv) Definitely yes, and (v) I don’t know.

#### Knowledge of ceremonial tobacco use

Student participants were asked to report their level of ceremonial tobacco knowledge by asking, “How much do you know about ceremonial tobacco?” with response options: (i) Nothing, (ii) Some, (iii) A lot, and (iv) Not sure.

#### Theory of planned behavior constructs

The theory of planned behavior (TPB) items were measured as, “If a friend offered me a puff of vape or a cigarette, it would be easy for me to refuse it,” and “It is mostly up to me whether I use commercial tobacco,” on a 5-point Likert scale ranging from Strongly Disagree (1) to Strongly Agree (5). Sample attitude-focused questions included questions like “Using nicotine/commercial tobacco (vaping, cigarettes, chew, etc.) is…” on a 5-point Likert scale ranging from Not Enjoyable (1) to Very Enjoyable (5). Sample perceived harm questions [13, 28] about nicotine use were assessed categorically with questions such as: “Do you think vaping is…” with the following answer options: (i) Very bad for one’s health, (ii) A little bad for one’s health, and (iii) Not bad for one’s health at all. Cronbach’s alpha coefficients were used to assess the internal consistency of the TPB items as a scale. Reliability estimates were low, with attitudes α = 0.377 at pre-test and α = 0.528 at post-test, subjective norms demonstrating α = 0.416 at pre-test and α = 0.389 at post-test, and perceived behavioral control demonstrating α = 0.141 at pre-test and α = 0.562 at post-test. Due to low reliability of the TPB items as a scale, the items were analyzed individually in the pre–post assessment analysis.

#### The TTM

To assess TTM stages of change [14], AI student participants were asked: “Which of the following statements best describes your attitude toward using nicotine/commercial tobacco products (e.g. cigarettes, e-cigarettes, vapes, JUULs, snuff, chew, dip, hookah) right now?” Response options ranged from (i) No intention to quit, (ii) Plans to quit, (iii) Recent quit attempts, or (iv) No use at all. To assess the “Intention” stage for vape use, participants rated the statement: “I will make an effort to not use nicotine/commercial tobacco (vaping, cigarettes, chew, etc.) products,” on a 5-point Likert scale ranging from Strongly Disagree (1) to Strongly Agree (5).

#### VE pilot intervention acceptability and satisfaction

Following each lesson, AI student participants completed open-ended questions that included prompts about which components they found most engaging and interesting, as well as key takeaways. A final satisfaction survey assessed student participants’ acceptability and satisfaction of the curriculum’s perceived helpfulness, applicability, and organizational quality on a scale of 1–10 (low to high).

#### Post-curriculum delivery reflection

The YAT participated in a final group reflection session to capture their perceived relevance, engagement, cultural resonance, and recommendations. This session assessed acceptability and satisfaction of the vaping pilot intervention from the youth leadership perspective to inform future AI-focused vaping curriculum development, refinement, and improvement.

### Statistical plan and analysis

Descriptive statistics were conducted for VE recruitment rates, student participant characteristics, baseline vape use, and satisfaction. For the pre–post assessment, Fisher’s exact test was conducted to analyze changes in students’ knowledge, perceived harms, and subjective norms. This test was chosen because it offers an exact *P*-value rather than relying on approximations, which may be incorrect given the small sample size [29]. The Wilcoxon Signed-Rank test was used to evaluate whether there were significant changes in the medians for attitudes, subjective norms, perceived behavioral control, and stages of change (intention). Similarly, the Wilcoxon Signed-Rank test was chosen because of the small sample size and non-normal distribution of the data [30]. Effect sizes were calculated using the number of valid paired responses for each item. By focusing on valid paired responses, the test assessed changes across the pre-test and post-test phases for scaled constructs. The SAS 9.3 software was utilized for the analyses and set at the alpha level of *P* < .05 and a 95% confidence interval. Average scores were calculated to assess student participant’s level of satisfaction based on their interest, perceived helpfulness, applicability, and organizational quality of the VE program. The open-ended feedback content from student participants’ final post-survey assessment and the YAT final reflection session were informally summarized in text format.

## Preliminary results

### Recruitment and retention rate

Of the 63 eligible AI student participants invited, 62 attended the informational session about the pilot intervention; one student was absent due to a sports commitment but returned to obtain a study flyer and consent packet the next day at the RISE center. Thirty-two AI students agreed to participate in the VE pilot intervention demonstrating a recruitment rate of 50.8% eligible students and 100% retention. Over half of the enrolled students were from RISE. Reasons for declining participation included concerns about privacy surrounding vape use or being labeled as a vape user (among AI student athletes specifically), challenges with completing assent and consent forms, and limited parental engagement particularly among those not involved in RISE programming.

### Participant characteristics

The majority of AI student participants identified as female (63%), 34% of students identified as male, and 3% identified as non-binary. The students were distributed across grades 7 through 12, with: 13% in 7th grade, 16% in 8th grade, 28% in 9th grade, 16% in 10th grade, 13% in 11th grade, and 16% in 12th grade.

### Vape use

American Indian student participants reported low commercial tobacco use, with 88% reporting none in the past 30 days, 3% reporting e-cigarette/vaping, 3% chewing tobacco, and other tobacco use (3%). Only 3% of the responses were missing. Flavored tobacco product use was rare, with 94% AI student participants reporting no flavored product use, and 6% reporting any flavored use (fruit/candy flavors and menthol products).

### Preliminary results for the pre–post assessment

#### Nicotine/vaping knowledge and perceived harms

At baseline assessment (pre-pilot intervention), the majority of AI student participants demonstrated high awareness of vape use across knowledge items (e.g. vapes containing nicotine, vape use targeted toward youth) (Table 2). Beliefs about tobacco companies marketing to youth statistically significantly increased from 94% to 97% (*P* = .001). A statistically significant increase in the proportion of AI students who perceived vaping is harmful to health increased from 97% to 100% agreement (*P* = .012) at post-pilot assessment. No other significant results were found for the remainder of the items on knowledge or perceived harms of vaping.

**Table 2:** *Vaping Endgame*’s preliminary pre–post assessment findings for nicotine knowledge, perceived harm, marketing strategies, and ceremonial tobacco use (*N* = 32).

Survey item	Pre-intervention			Post-intervention			*P*-value
	Agree *N* (%)	Disagree *N* (%)	Uncertain *N* (%)	Agree *N* (%)	Disagree *N* (%)	Uncertain *N* (%)	
**Knowledge**							
Vapes contain nicotine	30 (94)	2 (6)	–	32 (100)	–	–	.4921
Nicotine is addictive	32 (100)	–	–	30 (94)	2 (6)	–	.2736
Tobacco brands market to youth with bright colors and flavors	30 (94)	1 (3)	1 (3)	31 (97)	–	1 (3)	.001**
**Perceived harms**							
Cigarettes are harmful	31 (97)	1 (3)	–	32 (100)	–	–	1.00
Vaping (use) is harmful	28 (88)	4 (12)	–	32 (100)	–	–	.012*
Vapor is harmful	30 (94)	–	2 (6)	32 (100)	–	–	.383
**Knowledge of ceremonial tobacco use**	**Nothing/Unsure *N* (%)**	**Some *N* (%)**	**A lot *N* (%)**	**Nothing/Unsure *N* (%)**	**Some *N* (%)**	**A lot *N* (%)**	
How much do you know about ceremonial tobacco use?	13 (41)	17 (53)	2 (6)	7 (22)	20 (62)	5 (16)	.0015*

*N*, number; %, valid percent.

* *P* < .05.

** *P* < .001; columns with hyphens are empty values.

#### Ceremonial tobacco knowledge

Following the pilot intervention, AI student participants demonstrated a statistically significant increase in their knowledge of ceremonial tobacco. Initially, 41% indicated they knew “Nothing/Unsure,” 53% reported knowing “Some,” and 6% said “A lot.” At post-pilot assessment, these figures changed to 22% for “Nothing/Unsure,” 62% for “Some,” and 16% for “A lot” (*P* = .0015).

#### Tobacco brand marketing

At baseline assessment, 94% of AI student participants agreed that young people are targeted by tobacco brands using bright colors and flavors, and this increased significantly to 97% post-pilot intervention (*P* = .0012), indicating higher awareness of the marketing tactics toward youth from commercial tobacco industries.

### TPB measure changes

#### Attitudes

There was a statistically significant finding for an attitude item on the use of nicotine/commercial tobacco to manage stress (Table 3). The Wilcoxon Signed-Rank test indicated that AI students had higher agreement median scores that nicotine/commercial tobacco helps to manage stress at post-pilot assessment in comparison to the pre-pilot assessment (*Z* = −2.25; *P* = .02) with an effect size of *r* = −0.36. No other items for attitudes were significant for attitudes from pre-to-post pilot assessment.

**Table 3:** *Vaping Endgame*’s preliminary pre–post assessment findings for attitudes, subjective norms, perceived behavioral control, behavioral intention, and stages of change (*N* = 32).

Instrument and survey question	*Z*	*r*	*P*-value (two-tailed)
**TPB attitudes**			
“Using nicotine/commercial tobacco is enjoyable.”	−0.52	−0.09	.61
“Using nicotine/commercial tobacco helps manage stress.”	−2.25	−0.36	.02*
“Using nicotine/commercial tobacco is cool.”	−0.27	−0.05	.79
“Vaping is less addictive than cigarettes.”	−0.56	−0.10	.58
**Subjective norms**			
“Most people my age use nicotine/tobacco.”	−0.28	−0.05	.78
“It’s ok to use because friends/relatives use them.”	−0.89	−0.16	.37
“People important to me use nicotine/commercial tobacco.”	−1.28	−0.23	.20
**Perceived behavioral control**			
“It is mostly up to me whether I use commercial tobacco.”	−3.21	−0.50	.001**
“It is easy to get nicotine/commercial tobacco products.”	−2.14	−0.38	.032*
“Easy to refuse if friend offered puff of vape/cigarette.”	−1.02	−0.18	.307
**Behavioral intention**			
“I will make an effort not to use nicotine/commercial tobacco.”	−0.21	−0.04	.83
**The TTM stages of change (readiness)**			
“I do not plan to quit in the next 6 months.” “I plan to quit in the next 6 months.” “I plan to quit in the next 30 days.” “I have made a serious quit attempt in the past 6 months.” “I quit less than 6 months ago.” “I do not use nicotine/commercial tobacco products.”	−0.54	−0.10	.58

A Wilcoxon Signed-Rank test was conducted to evaluate changes in TPB constructs from the pre–post findings for this pilot intervention; The TTM measures are indicated as stages of change.

* *P* < .05.

** *P* < .001.

#### Perceived behavioral control

There was a statistically significant increase in perceived behavioral control following the pilot intervention. Results from the Wilcoxon Signed-Rank test indicated that AI students had higher median scores in the post-pilot assessment compared to pre-pilot assessment (*Z* = −3.21; *P* = .001) with a moderate effect size (*r* = −0.50) for the survey item, “It is mostly up to me whether I use commercial tobacco.”

#### Other measure changes

No other items (subjective norms, behavioral intention, stages of change) from pre- to post-test assessment were statistically significant. Tables 2 and 3 present all results for the items measured.

### Pilot intervention acceptability and satisfaction

#### Satisfaction ratings for VE

On a scale of 1–10 (low to high), AI student participants rated the VE curriculum highly across all four domains (lessons were interesting, perceived helpfulness, applicability, and organizational quality). Interest in the pilot curriculum and intervention was rated highly with an average score of 8.81 (SD = 2.02). The average score for the pilot intervention’s perceived helpfulness in supporting efforts to quit vaping was 8.84 (SD = 1.94). Student participants rated the pilot curriculum’s structure and organization with an average score of 8.87 (SD = 1.86), which was the same as the reported average score for the curriculum’s relevance and application to their lives (*M* = 8.87, SD = 1.91).

The open-ended feedback from the AI student participants at the end of the final lesson suggested that the pilot intervention was well received and culturally resonant. Survey reflections included takeaways about the risks of vaping with “I learned how addicting vaping can be” and appreciation for the cultural elements and teachings by highlighting that “Culture is important.” Student participants further expressed their appreciation for receiving a medicine bag. Students reported feeling safe in the classroom and expressed a strong sense of connection with the AI program facilitator.

#### Post-curriculum delivery reflection

YAT members found the curriculum to be “eye-opening,” especially in terms of understanding how vape product marketing is targeted toward youth. Members shared pride in leading the curriculum development, emphasizing that their input was accurately reflected. Suggestions for improvement included more ice-breakers during the sessions and a deeper integration of the pilot intervention within schools. YAT members stressed the value of youth leadership, stating young people are uniquely qualified to design effective peer interventions. They also highlighted the need for sustainable community initiatives in designing vaping pilot interventions to ensure ongoing positive impact.

## Discussion

This study described the AI cultural adaptation process for VE, a pilot vaping intervention and curriculum, highlighting its preliminary findings on pre–post outcomes, as well as AI student participant’s and YAT member’s satisfaction and acceptability. To our knowledge, this is the first contribution of a culturally responsive vaping pilot curriculum and intervention developed and delivered among AI students in middle and high school, suggesting potential for expansion and replication of future vaping interventions. To date, only one social media initiative in the United States called “Next Legends” is underway for AI youth but has yet to be evaluated [31]. The scarcity of AI culturally tailored vaping interventions makes our pilot intervention’s preliminary findings unique and timely in an urgent call to prevent vape use among AI youth. Guided by AI students, community members, and educators, the cultural adaptations to the INDEPTH curriculum and the applications of the Medicine Wheel showed alignment with AI values to support AI youth. The use of CBPR in the curriculum development process was critical in contributing to our 100% retention rate, building community trust, and ensuring that the pilot intervention reflected cultural values, beliefs, and priorities [18]. The integration of the PYD framework further supported a strength-based approach that fostered interactive engagement, cultural reflection, and open discussion between AI youth and the program facilitator.

Preliminary results from the pre–post assessment revealed a statistically significant increase in AI student participant’s perceived behavioral control, suggesting that culturally centered interventions may support meaningful change in vaping behaviors. Overall, attitudes toward e-cigarettes and nicotine were not significantly different, with the exception of a single item assessing beliefs about nicotine use as a coping mechanism for stress that had a bidirectional response. This item was intended to assess AI student participant’s existing attitudes of nicotine use for stress rather than to promote or endorse nicotine as a stress-management strategy. Although most AI students who participated reported no commercial tobacco/nicotine use, they expressed intentions to remain tobacco-free, which was one goal of this pilot intervention study.

Teaching AI student participants to distinguish the differences between ceremonial and commercial tobacco use had meaningful changes in knowledge, which studies have found to be critical for resisting the influences of commercial tobacco initiation [32]. This is an especially important preliminary finding given that commercial tobacco companies have historically targeted AI communities in stores and by mail by offering promotional vouchers for commercial tobacco [33]. Qualitative studies reveal that aggressive tobacco marketing influences AI youth’s decisions to vape, while motives not to vape included access to trusted adults and elders, suggesting that decision-making is underpinned by their cultures, context, and histories [34]. Strengthening cultural awareness through the Medicine Wheel may further counter these misconceptions and serve as protective factors against commercial nicotine/tobacco use.

Survey responses from the student participants and YAT reflections of the pilot intervention suggested positive indicators of acceptability, satisfaction, and cultural resonance. Student participants emphasized feeling a sense of safety receiving their medicine bag from an AI facilitator while YAT members expressed desire for more similar community-driven initiatives. These findings also point to the unique role of AI culture in fostering trust, engagement, and health-promoting behaviors among AI youth.

These preliminary findings may have direct implications for school administrators, educators, and researchers. School administrators and educators could adopt a health justice approach that may be used for vaping, as recommended by González *et al.* [35] that includes a three-prong framework: (i) addressing structural mechanisms that reinforce inequities; (ii) implement holistic, supportive interventions; and (iii) elevating frontline communities as leaders in designing and guiding solutions. Future research may also consider prospectively designing formal feasibility and evaluation studies using Bowen *et al.*’s [36] feasibility framework alongside validated implementation outcome measures such as the Acceptability of Intervention Measure and Feasibility of Intervention Measure [37] to more rigorously assess the scalability and broader implementation of culturally adapted vaping interventions for AI youth.

### Limitations

This study has several limitations to consider. The small sample size (*N* = 32) was drawn from a rural school district, which limits the generalizability of findings to other AI adolescents or various adolescent ethnic groups residing in similar regions. This study was not conducted as a formal feasibility study; therefore, findings should be considered in the context of preliminary pilot intervention insights that could inform feasibility studies. The pre–post assessment design did not include a control group for comparison, which presents challenges in attributing significant changes to the pilot intervention solely. Adaptations made to the measures using the TPB, TTM, and NYTS for measurement may not have captured all relevant constructs. In addition, Cronbach’s alpha scores on attitude, subjective norms, and perceived behavioral control as a scale were low, demonstrating that a more refined measurement framework is needed with a larger sample size for survey reliability in future vaping intervention studies among AI youth populations. The findings for items that were statistically significant reflected small shifts in response patterns (Tables 2 and 3). This is partly due to high baseline endorsement of the targeted beliefs, resulting in limited room for upward movement (i.e. ceiling effects). Future studies among AI youth with larger samples and more sensitive measures to better detect change at the upper end of belief distributions may help clarify these effects. AI student participants were mostly non-e-cigarette users and indicated high awareness of the perceived harms of vaping, which may have impacted the ability to detect vape use changes and contributed to the intervention’s non-significant findings. Nonetheless, there is value in the preliminary pre–post assessment findings, which demonstrated a change in perceived behavioral control and ceremonial tobacco knowledge, both of which are consistent with studies identifying key constructs and culturally grounded education serving as a positive predisposing and protective factors against commercial tobacco use [32, 38]. The low reported vaping rates among AI participants underscore the opportunity to pivot toward prevention, reaching youth outside of school disciplinary systems.

## Conclusion

The marketing landscape of nicotine and vape products continues to evolve and regulations on flavored tobacco/nicotine products may not fully prevent AI youth from using commercial tobacco (vape); however, we propose that community involvement can complement changing regulations while acknowledging the ceremonial use of tobacco, as shown in this study. The contribution of our pilot intervention curriculum to this space offers a community-driven and school-embedded model. Preliminary findings suggest the need for continued investment in educational vaping interventions that empower AI students’ perceived behavioral control while acknowledging cultural practices of ceremonial tobacco. Our pilot intervention design promotes the transition to a supportive approach guided by PYD and CBPR frameworks for AI students who may be vulnerable to vaping. Finally, our study’s approach may help cultivate a holistic educational setting for vaping prevention and strengthen trust among youth, AI community leaders, school faculty, and staff.

## Funding sources

This study was funded by the Tobacco-Related Disease Research Program (Grant Number: TR32CR4887).

## Involvement of the funder

The funders had no role in the design and conduct of the study; collection, management, analysis, and interpretation of the data; preparation, review, or approval of the manuscript; or decision to submit the manuscript for publication.

## Data Availability

De-identified data from this study are not available in a public archive. De-identified data from this study will be made available (as allowable according to institutional IRB standards) by emailing the corresponding author. At the end of the grant period, the data will be placed in an NIH-approved data repository.

## Welfare of animals

This article does not contain any studies with animals performed by any of the authors.
